# Focused ultrasound for the treatment of glioblastoma

**DOI:** 10.1007/s11060-022-03974-0

**Published:** 2022-03-10

**Authors:** Jill W. Roberts, Lauren Powlovich, Natasha Sheybani, Suzanne LeBlang

**Affiliations:** 1 Focused Ultrasound Foundation, 1230 Cedars Court, Suite 206, Charlottesville, VA 22903 USA; 2 Department of Biomedical Engineering, University of Virginia, Charlottesville, VA 22908 USA

**Keywords:** Glioblastoma, Focused ultrasound, Blood–brain barrier opening, Tumor ablation, Histotripsy, Cancer immunotherapy, Sonodynamic therapy

## Abstract

**Purpose:**

Six years ago, in 2015, the Focused Ultrasound Foundation sponsored a workshop to discuss, and subsequently transition the landscape, of focused ultrasound as a new therapy for treating glioblastoma.

**Methods:**

This year, in 2021, a second workshop was held to review progress made in the field. Discussion topics included blood–brain barrier opening, thermal and nonthermal tumor ablation, immunotherapy, sonodynamic therapy, and desired focused ultrasound device improvements.

**Results:**

The outcome of the 2021 workshop was the creation of a new roadmap to address knowledge gaps and reduce the time it takes for focused ultrasound to become part of the treatment armamentarium and reach clinical adoption for the treatment of patients with glioblastoma. Priority projects identified in the roadmap include determining a well-defined algorithm to confirm and quantify drug delivery following blood–brain barrier opening, identifying a focused ultrasound-specific microbubble, exploring the role of focused ultrasound for liquid biopsy in glioblastoma, and making device modifications that better support clinical needs.

**Conclusion:**

This article reviews the key preclinical and clinical updates from the workshop, outlines next steps to research, and provides relevant references for focused ultrasound in the treatment of glioblastoma.

## Introduction

**Fig. 1:**
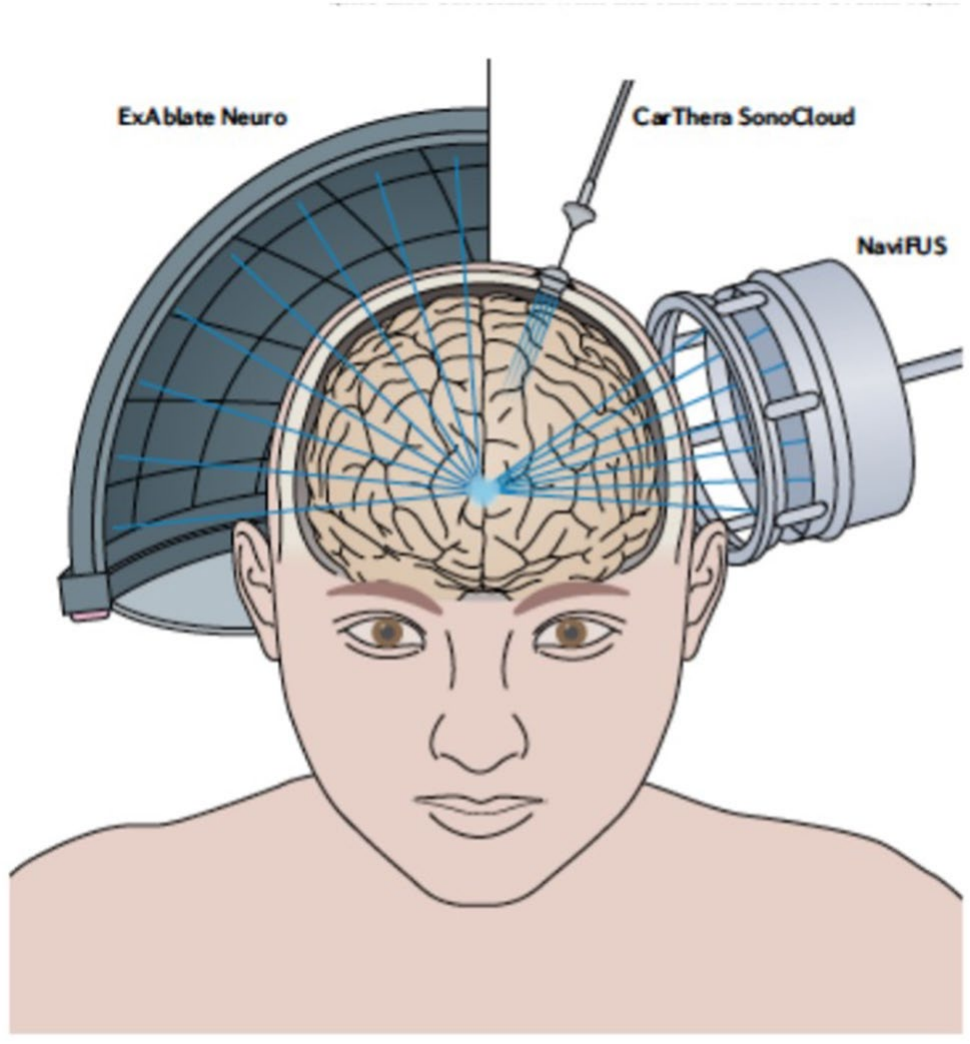
Focused Ultrasound Devices for GBM Treatment. The ExAblate Neuro device uses MRI images during the procedure to guide the focused ultrasound beam. The NaviFUS system is guided by neuronavigation with a previously performed MRI scan to guide the beam to the target region; it can be performed in an office setting. The CarThera SonoCloud device is implanted in the skull overlying the target region and does not use imaging guidance. Image used with Copyright Clearance Center permission from Meng Y, Hynynen K, Lipsman N, *Nat Rev Neurol*. 2021;17(1):7–22 [46]

**Fig. 2:**
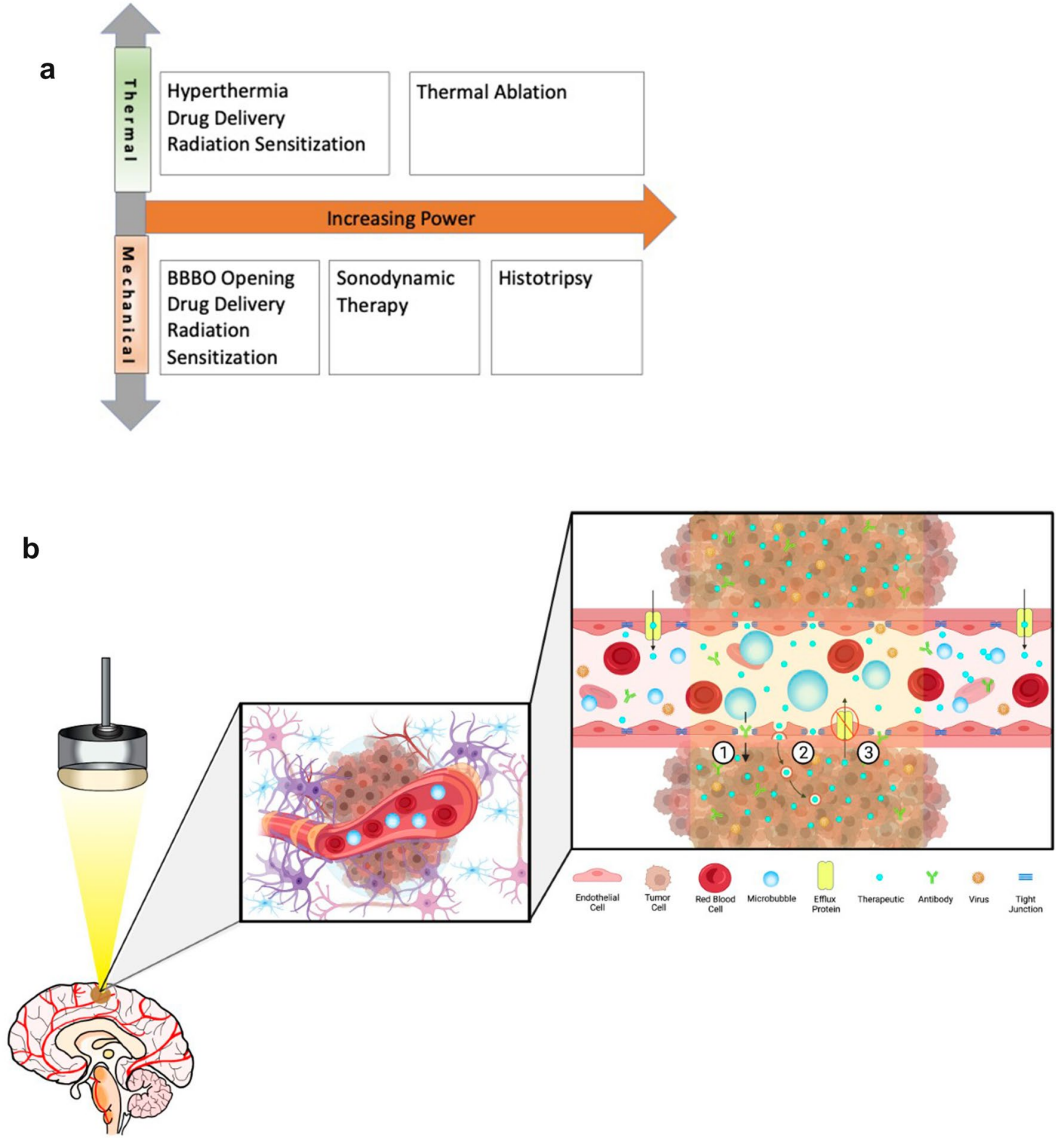

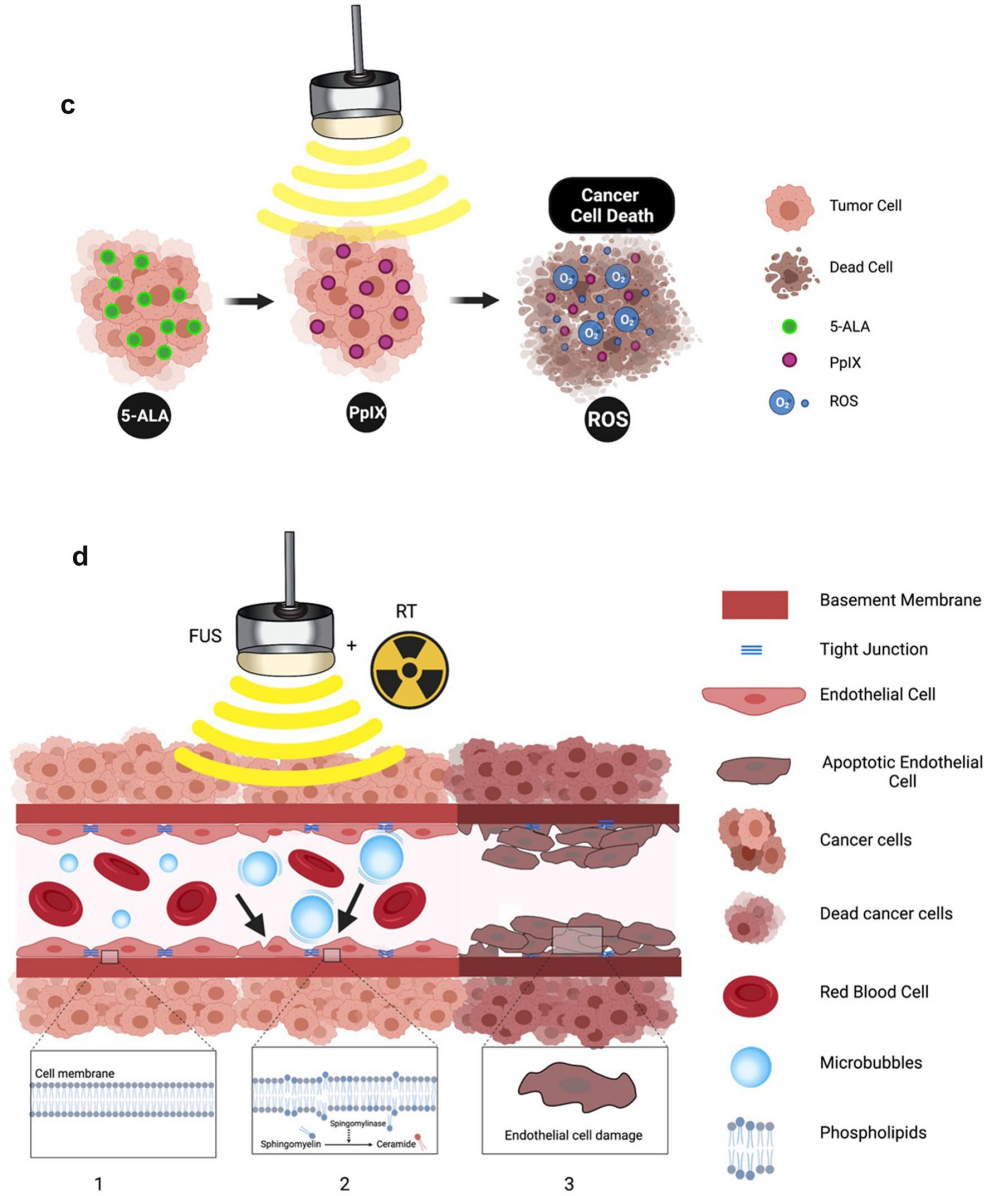
Thermal and Mechanical Focused Ultrasound Mechanisms of Action include Blood–Brain Barrier Opening, Sonodynamic Therapy, Radiation Sensitization, Histotripsy, and Liquid Biopsy. **A** Various thermal and mechanical focused ultrasound mechanisms of action related to power. **B** Blood–Brain Barrier Opening for Drug Delivery. In the precise location of the focused ultrasound beam, intravenously injected intravenous microbubbles expand and contract causing: 1. Opening of the tight junctions; 2. An increase in the number of transcytotic vesicles; and 3. Decreasing efflux pumps, which contribute to enhanced therapeutic delivery of chemotherapies, antibodies, or cargo-loaded viral particles across the BBB. **C** Sonodynamic Therapy. An intravenously injected sonosensitizer (5-ALA) crosses the blood–brain barrier, accumulates in tumor cells, and converts to protoporphyrin IX (PpIX). Focused ultrasound activates the PpIX, which generates reactive oxygen species (ROS). ROS causes apoptosis and tumor cell death. **D** Radiation Sensitization. In the presence of microbubbles, the focused ultrasound beam causes oscillations of injected intravenous microbubbles, which mechanically damages the endothelium (black arrows) and releases ceramide. Along with radiation therapy, ceramide induces endothelial and tumor cell death

Focused ultrasound is an early stage, therapeutic technology that offers possible adjuvant or alternative treatment strategies for glioblastoma (GBM). In May 2021, the Focused Ultrasound Foundation (www.fusfoundation.org) convened its second two-day GBM workshop to engage critical stakeholders, including researchers, clinicians, industry, government, and others, to share and combine knowledge to advance the field. The technology has made great advancements since the first workshop on this topic, which was held in 2015 [1]. For example, 13 clinical trials (Table 1) with three manufacturers (Fig. 1) at 20 centers worldwide are currently underway. Before the virtual workshop, experts recorded 23 educational talks that were made available to attendees one week in advance of the live event. These recordings described several different focused ultrasound mechanisms of action that are in various stages of research to treat GBM (Fig. 2A). During the workshop, attendees addressed burning questions within key topic areas through in-depth and interactive expert panel discussions. This article reviews the key preclinical and clinical updates from the workshop, outlines next steps to research, and provides relevant references for focused ultrasound in the treatment of GBM.

**Table 1:** Clinical Trials Using Focused Ultrasound for Blood–Brain Barrier Opening, Sonodynamic Therapy, and Radiation Sensitization for the Treatment of Glioblastoma

A. Blood–Brain Barrier Opening Clinical Trials (n = 10)						
Title	ClinicalTrials.gov NCT number	Location (sites)	Enrollment	Device	Status	Drug
ExAblate Blood–Brain Barrier Disruption (BBBD) for Planned Surgery in Suspected Infiltrating Glioma	03322813	USA	15	InSightec ExAblate	Active, not recruiting	None, planned surgical resection
Assessment of Safety and Feasibility of ExAblate Blood–Brain Barrier (BBB) Disruption	03551249	USA (4)	20	InSightec ExAblate	Recruiting	Adjuvant TMZ
Assessment of Safety and Feasibility of ExAblate Blood–Brain Barrier (BBB) Disruption for Treatment of Glioma	03616860	Canada	20	InSightec ExAblate	Recruiting	TMZ maintenance
ExAblate Blood–Brain Barrier Disruption for Glioblastoma in Patients Undergoing Standard Chemotherapy	03712293	Republic of Korea	10	InSightec ExAblate	Recruiting	Adjuvant TMZ
Safety and Efficacy of Transient Opening of the Blood–brain Barrier (BBB) With the SonoCloud-9	03744026	USA (2); France (4)	33	Carthera SonoCloud-9	Active, not recruiting	Carboplatin
ExAblate Blood–Brain Barrier Disruption for the Treatment of rGBM in Subjects Undergoing Carboplatin Monotherapy	04417088	USA (4)	30	InSightec ExAblate	Recruiting	Carboplatin
ExAblate Blood–Brain Barrier Disruption with Carboplatin for the Treatment of rGBM	04440358	Canada (1), South Korea (1)	50	InSightec ExAblate	Recruiting	Carboplatin
Efficacy and Safety of NaviFUS System add-on Bevacizumab (BEV) in Recurrent GBM Patients	04446416	Taiwan (1)	10	NaviFUS	Recruiting	Bevacizumab
Ultrasound-based Blood–brain Barrier Opening and Albumin-bound Paclitaxel for Recurrent Glioblastoma (SC9/ABX)	04528680	USA (1)	39	CarThera SonoCloud-9	Recruiting	Albumin-bound paclitaxel
SonoCloud-9 Device for Blood–Brain Barrier Opening in First Line Temozolomide Glioblastoma Patients	04614493	Belgium (1), France (5), Switzerland (1)	66	CarThera SonoCloud-9	Recruiting	Adjuvant TMZ

B. Sonodynamic Therapy (n = 2) and Radiation Sensitization (n = 1) Clinical Trials						
Title	ClinicalTrials.gov NCT number	Location (sites)	Enrollment	Device	Status	Agent
Study of Sonodynamic Therapy in Participants With Recurrent High-Grade Glioma	04559685	USA (1)	30	Insightec ExAblate	Recruiting	5-ALA (intravenous) SONALA-001
Sonodynamic Therapy With ExAblate System in Glioblastoma Patients (Sonic ALA)	04845919	Italy (1)	5	Insightec ExAblate	Not yet recruiting	5-ALA (oral)
Evaluate the Safety and Preliminary Efficacy of the Combination of NaviFUS System with Re-irradiation for rGBM Patients	04988750	Taiwan (1)	8	NaviFUS	Recruiting	Radiation sensitization

TMZ, temozolomide

## Blood–brain barrier opening

The blood–brain barrier (BBB) is an obstacle to the effective treatment of brain tumors with therapeutic agents. Although there are some portions of gliomas that display magnetic resonance imaging (MRI) contrast enhancement suggesting “leaky” vessels that allow extravasation of large molecules, it is known that tumor cells extend far beyond the borders of these enhancing areas; therefore, the intact BBB remains an obstacle in the effective delivery of anti-cancer therapies [2]. When combined with microbubble administration, low-intensity focused ultrasound is a promising technique for BBB opening (BBBO) that has been widely studied across preclinical models with encouraging results [3–5] (Fig. 2B). Early-stage clinical trials have also shown safety and feasibility for this technique [6–9].

### Microbubble administration protocol

Microbubbles are an essential component for focused ultrasound–induced BBBO, because the focused ultrasound makes them oscillate, and this vibration creates penetrable gaps in the BBB’s tight endothelial junctions. Various microbubbles have been used for focused ultrasound–induced BBBO, including commercial ultrasound contrast agents and bubbles specifically designed for focused ultrasound BBBO. Agents that are designed for BBBO should (i) easily reach stable cavitation with ultrasound, (ii) have in vivo stability in the blood circulation, and (iii) have shelf stability for storage purposes [10, 11]. The most commonly used commercially available microbubbles for focused ultrasound BBBO are Definity™ (Lantheus Medical Imaging), SonoVue™ (Bracco), Optison™ (GE Healthcare) and Sonazoid™ (GE Healthcare). In a prerecorded talk, Francesco Prada, MD, reviewed various microbubble administration protocols, which can be safely administered via intravenous bolus injection or continuous infusion. More data are needed to conclude the optimal administration protocol [12, 13]. An expert panel featuring Nathan McDannold, PhD, Francesco Prada, MD, Elisa Konofagou, PhD, and Kullervo Hynynen, PhD, discussed this conundrum and shared that, in clinical trials, continuous infusion of microbubbles is being used for safety reasons, but most preclinical work was carried out using bolus injections. There was enthusiastic consensus that a microbubble should be designed specifically for focused ultrasound–induced BBBO.

### Sonication parameters

Several focused ultrasound parameters should be considered for safely and effectively inducing BBBO. The sonication parameters used affect the depth of ultrasound penetration and the duration of opening. Nathan McDannold, PhD, reviewed these variables and their common values (Table 2). Other factors that might affect the degree of BBBO are anesthesia, supplemental oxygen, steroid administration, and timing of focused ultrasound in relationship to microbubble administration. Dr. McDannold explained that the penetration depth and the duration of opening is affected by the sonication parameters used. Panelists Nathan McDannold, PhD, Francesco Prada, MD, Elisa Konofagou, PhD, and Kullervo Hynynen, PhD, agreed that there are no well-defined *optimal* sonication parameters for BBBO; ideally, the parameters used for each patient will lead to maximum drug delivery with minimal tissue damage. They agreed that the best focused ultrasound settings for inducing BBBO will vary based on the size of the molecule being delivered and the type of microbubble being used.

**Table 2:** Focused ultrasound parameters for inducing blood–brain barrier opening

Variable	Typical values
Pressure amplitude	< 1 MPa
Frequency	200–700 kHz
Burst length	1–10 ms
Pulse repetition frequency	1–10 Hz
Duration of sonication	60–120 s
Microbubble agent	Definity, Sonovue, Optison
Microbubble dose	Fraction of clinical dose to 100 × clinical dose

### Confirmation of BBBO

After microbubble administration and the application of low-intensity focused ultrasound to the target area, the desired effect of increased permeability of the BBB must be confirmed. Michael Canney, PhD, Nathan McDannold, PhD, Antonis Pouliopoulos, PhD, and Raag Airan, MD, PhD, discussed methods used to confirm BBBO [14, 15]. There was consensus that the most common methods to confirm BBBO are T1-weighted and dynamic contrast-enhanced (DCE) MR imaging, especially in clinical studies. Other methods used, mostly in preclinical models, include fluorescent tracers, mass spectrometry, acoustic backscatter, and passive acoustic mapping. Although MRI works well to confirm increased leakiness of the BBB, better imaging modalities must be investigated to quantify or confirm that the desired drug is reaching the target area in increased concentrations.

## Targeted drug delivery across the BBB

Four presentations highlighted clinical trials involving targeted drug delivery with focused ultrasound–induced BBBO. Nir Lipsman, MD, PhD, Graeme Woodworth, MD, Adam Sonabend, MD, and Ko-Ting Chen, MD, each shared their Phase I and II clinical trial experience. To date, the safety and feasibility of focused ultrasound–induced BBBO has been established with three different clinical devices: InSightec ExAblate, CarThera Sonocloud, and NaviFUS (Fig. 1). Ongoing clinical trials continue to examine safety, feasibility, and outcome data using various chemotherapeutics (Table 1).

The panel discussion moderated by Nir Lipsman, MD, PhD, included Jin Woo Chang, MD, PhD, Alexandra Golby, MD, Adam Sonabend, MD, Roger Stupp, MD, and Graeme Woodworth, MD, who raised important unanswered questions surrounding ongoing and future clinical trials. Most of the current clinical trials are treating patients with recurrent GBM, but there should be more consideration for upfront treatment when there is less tumor heterogeneity. Outcome measures should include progression-free survival and overall survival. Previous studies have shown that greater doses of temozolomide (TMZ) do not affect outcome, so increasing the concentration of TMZ at the site of BBBO is not likely to increase efficacy [16]. A control arm is important to include in focused ultrasound–induced BBBO clinical studies, and Bayesian-designed studies to simultaneously evaluate multiple therapies should be explored.

### Delivery of immuno-therapeutics across the BBB

Manmeet Ahluwalia, MD, moderated a panel that included John de Groot, MD, Amy Heimberger, MD, and Patrick Wen, MD, who commented on focused ultrasound–enhanced delivery of immunotherapeutic agents to brain tumors. Immunotherapy has become a pillar of cancer therapy, yet GBMs have yet to be impacted by immunotherapeutics that have helped treat other cancers. Focused ultrasound is a promising technology to potentiate immunotherapeutics [17]. No clinical trials are currently using combination immunotherapy with focused ultrasound, but the group suggested the following therapeutics for GBM therapy during BBBO: immune checkpoint-directed antibodies, adoptive T cells, natural killer (NK) cells, chimeric antigen receptor T cells, and genetically modified antigen-presenting cells. The panel agreed that additional preclinical work was needed, but that it may be too risky to base a large, phase III trial off preclinical data, because mouse models do not sufficiently recapitulate human GBM. Neoadjuvant trials prior to surgery to study whether combination with focused ultrasound could evoke the desired response might be a better approach. Additionally, Dr. Heimberger advised that performing pathology on a small sample of the tumor could provide misleading results because it is unknown whether there is uniform immune cell dispersal throughout the tumor microenvironment (i.e., in GBM, T cells are limited to the perivascular space).

## Immunomodulation

Kelsie Timbie, PhD, presented a prerecorded talk on the current state of focused ultrasound immunomodulation research. She provided a review of the literature [18–24] and described the Foundation’s first multisite GBM consortium project, which began in 2016, to investigate the effects of different focused ultrasound modalities on the immune system and cancer immunity cycle. Each of the seven participating centers used the same animal model of GBM, and results varied. The group using thermal ablation was the only one to achieve tumor growth control. Thermal ablation did not achieve an immune response in the tumor, but hyperthermia increased infiltration of activated NK, effector CD8 cells, and myeloid-derived suppressor cells (MDSC). In contrast, mechanical ablation with histotripsy increased proliferation of CD3-positive, CD4-positive, and CD8-positive T cells. Histotripsy also enhanced dendritic cell activation, decreased MDSCs, and increased interferon gamma production. Overall, the main takeaway was that different focused ultrasound mechanisms have vastly different effects on the immune system.

During the panel discussion, moderator Michael Lim, MD, and panelists Costas Arvanitis, PhD, Timothy Bullock, PhD, Theresa LaVallee, PhD, and Tao Sun, PhD, described several of these immune responses to focused ultrasound and discussed ways to monitor them. Future research suggestions included considering the intersection of focused ultrasound–enhanced immunomodulation with GBM lymphatics and more exactly determining the role of focused ultrasound for GBM immunomodulation. Additional studies are needed to understand the interplay between the various focused ultrasound mechanisms and immunomodulation. New projects could investigate whether focused ultrasound can induce trafficking and activation of immune cells and explore whether focused ultrasound can activate microglia and whether this microglia activation is beneficial in GBM.

## Other mechanisms of action

Other focused ultrasound mechanisms of action, such as sonodynamic therapy (SDT), radiation sensitization, and histotripsy have been investigated in preclinical models and are emerging in clinical trials.

### SDT

Francesco Prada, MD, discussed “Sonodynamic Therapy: Concept, Mechanisms, and Application to Brain Cancer” in his prerecorded presentation (see Resources section below). Sonodynamic therapy involves activating a sonosensitizing agent (e.g., 5-ALA, fluorescein) with ultrasound, which results in the creation of reactive oxygen species leading to cell death. Sonosensitizers are chemical compounds that selectively accumulate in tumor cells, such as glioblastomas, and are currently used to guide surgical resection because they are also activated by light, which allows for improved intraoperative visualization of the tumor [25]. Panel moderator Jason Sheehan, MD, PhD, and panelists Kullervo Hynynen, PhD, Hao-li Liu, PhD, Stuart Marcus, MD, PhD, and Francesco Prada, MD, all agreed that preclinical data support SDT as a potential treatment for GBM. Although the exact mechanism by which focused ultrasound activates 5-aminolevulinic acid (5-ALA) is not definitively understood, ablation of GBM tumor models due to apoptosis (Fig. 2C) has been achieved in preclinical studies, as evidenced by MRI and histologic evaluation [26–28]. Various sonosensitizers, such as 5-ALA and fluorescein, preferentially accumulate in tumoral tissue. Based on the in vivo study on large animals, there is no damage identified to the normal brain with the maximum 5-ALA dose of 100 mg/kg body weight and fluorescein dose of 20 mg/kg body weight, respectively. Intravenous 5-ALA formulation may prove safer than the oral route, because it bypasses the stomach and liver, preventing side effects (e.g., nausea, vomiting) and diminishing changes in liver function tests. In addition, the intravenous formulation may allow for more efficient delivery of 5-ALA to the tumor. There is a current clinical trial (NCT 04559685) using intravenous 5-ALA with focused ultrasound for SDT of GBM in the United States and another study (NCT 04845919) that is using oral 5-ALA in Italy (Table 2).

### Radiation sensitization

A prerecorded lecture by Frederic Padilla, PhD, the “Role of Focused Ultrasound for Radiosensitization of GBM,” (see Resources section below) provided an overview of the proposed mechanisms of action for how focused ultrasound causes radiosensitization of tissues. Because resistance to radiation therapy results from tumor hypoxia [29], increasing blood flow and oxygenation could increase radiation sensitization. Various modes of focused ultrasound, such as mild hyperthermia, can increase oxygenation and perfusion [30, 31], and nonthermal effects from focused ultrasound and microbubbles combined to open the BBB have been reported to also improve oxygenation and recruit immune cells (see Chia-Jung Lin brain tumor oral presentation and Hao-Li Liu brain tumor panel at the 2020 Focused Ultrasound International Symposium) [32]. Focused ultrasound and microbubbles can also cause vascular shut down, leading to complete anoxia and contributing to tumor cell death downstream (Fig. 2D) [33, 34]. Greg Czarnota, MD, PhD, moderated a discussion with panelists Hao-Li Liu, PhD, and Frederic Padilla, PhD. The first-in-human clinical trial using the NaviFUS focused ultrasound system for radiosensitization of GBM (NCT 04988750) is now recruiting in Taiwan.

### Histotripsy

Zhen Xu, PhD, moderated a discussion on histotripsy as a form of mechanical focused ultrasound ablation. Panelists Tatiana Khoklova, PhD, Joan Vidal-Jove, MD, PhD, and Eli Vlaisavljevich, PhD, provided prerecorded videos on histotripsy for brain applications (see Resources section below). Histotripsy uses focused ultrasound to mechanically destroy tissue, similar to lithotripsy, and can target brain tissue and tumors in a more well-defined region and faster than focused ultrasound thermal ablation with the added benefit of avoiding skull heating. In animal models, some swelling and bleeding have been noted following intracranial histotripsy; therefore, beam parameters (e.g., frequency, number of sonications, treatment time) need to be optimized to avoid complications [35]. Early preclinical results suggest that histotripsy may elicit an immune response. For example, mouse studies in GBM models treated with focused ultrasound released tumor antigens and recruited and activated immune cells, changing the tumor microenvironment from cold to hot [36]. To date, there are no clinical trials with histotripsy for brain tumors.

## Technology

Although three clinically available focused ultrasound devices are currently being used for focused ultrasound–induced BBBO in GBM, additional clinical devices are at various stages of development at three institutions. During the workshop, the expert panel on technology gaps and desired features and functionalities was moderated by Elisa Konofagou, PhD, and included Kullervo Hynynen, PhD, Ying Meng, MD, PhD, Graeme Woodworth, MD, and Fred Wu, MD, PhD. The group listed several desired focused ultrasound system improvements, including the ability to expand the treatment envelope so that a greater tumor volume or a two-centimeter margin of tissue around a resection cavity could be treated. They added that brain treatment head frames should be more patient friendly, comfortable, and customized for each skull and tumor location. The current head frames being used for MRI-guided focused ultrasound BBBO are pinned stereotactic head frames that were designed for radiosurgery. Although complete stillness and precision is required for focused ultrasound thermal ablation of brain targets, a less invasive and more comfortable headframe could be designed for the distinct needs of BBBO procedures. Avoiding the need for head shaving is also important for patients. Accurate mathematical modeling algorithms are needed for treatment prediction, and a quality assurance process is needed.

## Treatment monitoring

Various imaging modalities and liquid biopsy (LB) are being developed for use in treatment monitoring in focused ultrasound GBM applications.

### Radiologic assessment

Benjamin Ellingson, PhD, provided an overview lecture on imaging modalities for GBM monitoring (see Resources below). The modified Response Assessment Neuro Oncology (RANO) criteria is used to evaluate for tumor growth versus pseudo progression and is especially helpful for longitudinal follow-up during adaptive clinical trials to mitigate false negative and false positive results [37]. Other advanced techniques to quantify vascular permeability with focused ultrasound studies include DCE MRI [38] and contrast-enhanced (CE) T1 digital subtraction maps [39]. Dynamic susceptibility contrast (DSC) MRI also allows for quantification of blood volume, flow, and vessel size [40, 41]. These imaging studies can be incorporated into clinical trials to evaluate mechanisms of action.

Patrick Wen, MD, moderated a discussion with Benjamin Ellingson, PhD, Ali Nabavizadeh, MD, and Max Wintermark, MD, as panelists. Various challenges still exist while evaluating MR imaging studies for extent of tumor infiltration, treatment response, and differentiating progression from pseudo progression. Positron emission tomography scanning with varying tracers has contributed to our understanding of tumor metabolism and can be used to understand how focused ultrasound works and help differentiate progression from pseudo progression. In the future, machine learning and artificial intelligence will assist in evaluating subtle changes in imaging findings during treatment monitoring.

### Liquid biopsy (LB)

**Fig. 3:**
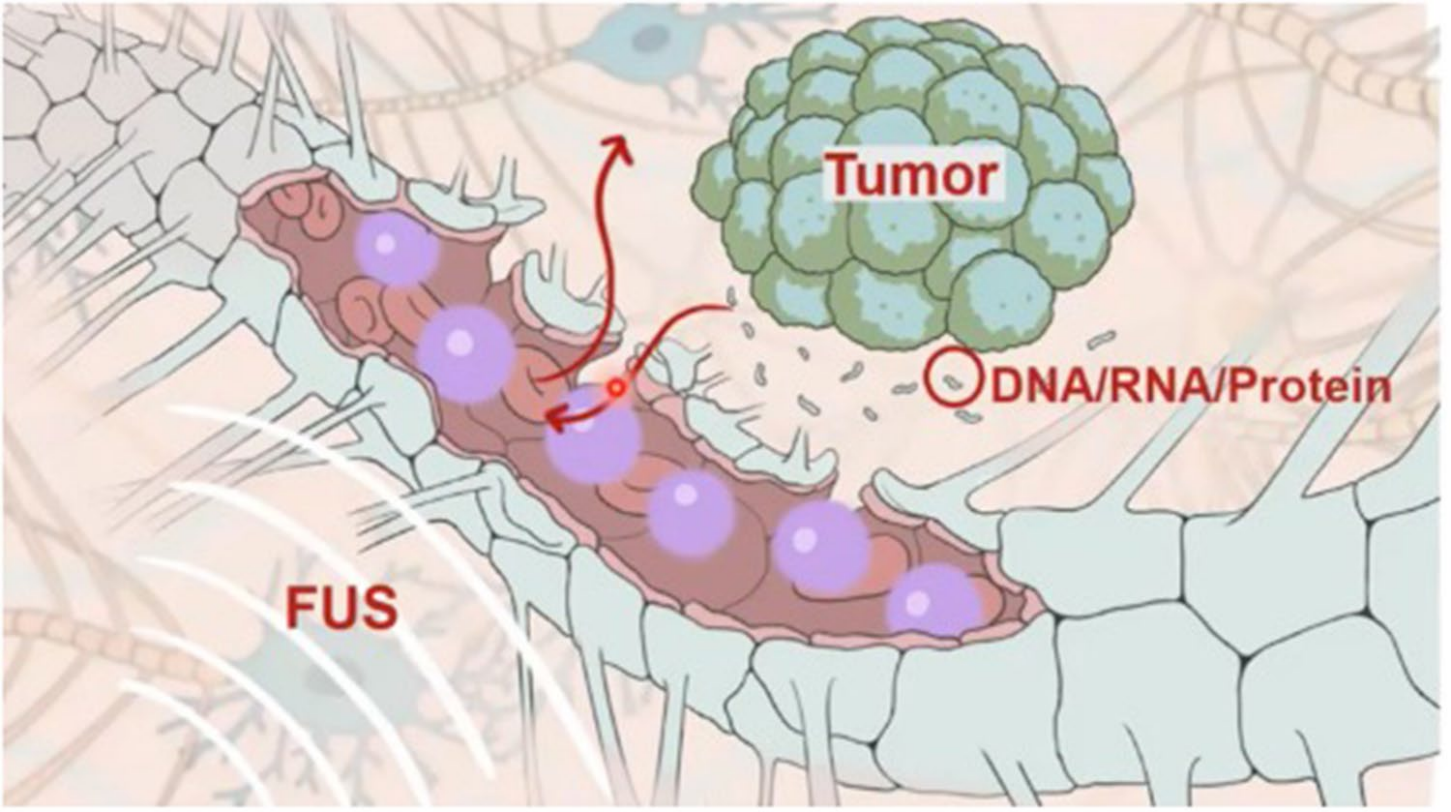
Blood–Brain Barrier Opening for Liquid Biopsy. Blood–brain barrier opening with focused ultrasound (FUS) allows bidirectional flow of molecules. DNA, RNA, and protein tumor markers enter the peripheral circulation, where they can be collected and analyzed. Unpublished image courtesy of Hong Chen, PhD, Washington University in St. Louis

Ying Meng, MD, PhD, summarized the current knowledge of focused ultrasound–enabled LB for brain tumors (see Resources below). Typically, LB for brain tumors has been limited due to the lower amounts of circulating tumor DNA compared to systemic cancers. This is likely due to decreased flow across the BBB. Several preclinical publications demonstrated the ability to detect analytes from the brain in the peripheral circulation after focused ultrasound–induced BBBO, detailing the possibility of bidirectional flow across the BBB for creating a “window” into the brain (Fig. 3—reprinted with permission from Chen, et. al) [31, 42, 43]. Dr. Meng presented the results of first-in-human data, revealing an increase in the amount of cell-free DNA and neural derived extracellular vesicles released into the peripheral blood after focused ultrasound–induced BBBO to improve temozolomide delivery in clinical trial patients with GBM [44]. In addition, the analytes had a distinct signature with more hypermethylation, making them likely derived from glial and neuronal tissue.

Moderator Chetan Bettegowda, MD, PhD, and panelists Hong Chen, PhD, Ying Meng, MD, and Houtan Noushmehr, PhD, discussed numerous technical issues, such as when to obtain LB after focused ultrasound–induced BBBO, what volume of tissue should be targeted, and which sonication parameters are optimal and how they will vary depending on the size of the analyte. There was consensus regarding the promise of focused ultrasound–induced LB to be used as a progression monitoring tool and to discern progression from pseudo progression. A specific advantage of focused ultrasound is its ability to spatially target a discrete region of the tumor or peritumoral tissues to interrogate the analytes in that specific region.

## Clinical trial design

Gautam Mehta, MD, and Gregory Clement, PhD, provided the US Food and Drug Administration perspective on how oncology trials are monitored and described regulatory considerations for conducting combination trials using focused ultrasound. Jessica Foley, PhD, moderated a panel discussion that included Amy Barone, MD, Bennet Blumenkopf, MD, Greg Clement, PhD, Subha Maruvada, PhD, Gautam Mehta, MD, and Matthew Myers, PhD, who commented on on what the FDA requires for drug-device combination procedures and those that combine the use of microbubbles and focused ultrasound. The FDA considers both microbubbles and focused ultrasound devices. A lively discussion covered several aspects of this type of clinical trial design. Evidence for reimbursement, another important aspect of clinical trial design, was discussed by Stephanie Kennan, MBA, and Dee Kolanek, AAS, and moderated by Jessica Foley, PhD. The workshop’s white paper includes more information on these topics [45].

## Future directions

The Focused Ultrasound Foundation’s second glioblastoma workshop convened an expert group of clinicians and scientists to share the most current preclinical and clinical advancements in the field of focused ultrasound for GBM. The most advanced application is focused ultrasound–induced BBBO to enhance the delivery of therapeutics to brain tumors, with several ongoing clinical trials. Gaps still exist with this therapy, and most notably include the lack of a well-defined algorithm to confirm and quantify drug delivery following BBBO and a lack of generalizable knowledge on the effect that the type and administration route of microbubbles has on BBBO. These gaps have been identified, and the Foundation is actively working with teams of researchers to address them.

In addition to focused ultrasound–induced BBBO, other promising mechanisms are beginning to gain traction for treating brain tumors. SDT and histotripsy are among the newer focused ultrasound techniques that have particularly encouraging outlooks. Focused ultrasound–enhanced LB is another promising application. The Foundation is dedicated to leveraging its resources to strengthen the science, encourage collaboration, and move these applications forward to improve the lives of patients with GBM as soon as possible.

If you have research or research questions that align with the Focused Ultrasound Foundation’s mission and would like to speak to one of the Brain Tumor Program leads, please contact Lauren Powlovich or Suzanne LeBlang or submit an abstract for funding at: https://www.fusfoundation.org/the-foundation/programs/research.

## Data Availability

Data sharing is not applicable to this article. No datasets were generated or analyzed.
